# Atomistic deformation mechanism of silicon under laser-driven shock compression

**DOI:** 10.1038/s41467-022-33220-0

**Published:** 2022-09-21

**Authors:** Silvia Pandolfi, S. Brennan Brown, P. G. Stubley, Andrew Higginbotham, C. A. Bolme, H. J. Lee, B. Nagler, E. Galtier, R. L. Sandberg, W. Yang, W. L. Mao, J. S. Wark, A. E. Gleason

**Affiliations:** 1 SLAC National Accelerator Laboratory, 2575 Sand Hill Rd., Menlo Park, CA 94025 USA; 2 Department of Physics, Clarendon Laboratory, Univeristy of Oxford, Parks Road, Oxford, OX1 3PU UK; 3 Department of Physics, University of York, Heslington York, YO10 5DD UK; 4 Los Alamos National Laboratory, Los Alamos, NM 87545 USA; 5 Center for High Pressure Science and Technology Advanced Research (HPSTAR), Shanghai 201203, China; 6 Geological Sciences, Stanford University, 367 Panama St., Stanford, CA 94305 USA; 7 Present Address: Department of Physics and Astronomy, Brigham Young University, Provo, UT 84602 USA

**Keywords:** Phase transitions and critical phenomena, Structure of solids and liquids

## Abstract

Silicon (Si) is one of the most abundant elements on Earth, and it is the most widely used semiconductor. Despite extensive study, some properties of Si, such as its behaviour under dynamic compression, remain elusive. A detailed understanding of Si deformation is crucial for various fields, ranging from planetary science to materials design. Simulations suggest that in Si the shear stress generated during shock compression is released via a high-pressure phase transition, challenging the classical picture of relaxation via defect-mediated plasticity. However, direct evidence supporting either deformation mechanism remains elusive. Here, we use sub-picosecond, highly-monochromatic x-ray diffraction to study (100)-oriented single-crystal Si under laser-driven shock compression. We provide the first unambiguous, time-resolved picture of Si deformation at ultra-high strain rates, demonstrating the predicted shear release via phase transition. Our results resolve the longstanding controversy on silicon deformation and provide direct proof of strain rate-dependent deformation mechanisms in a non-metallic system.

## Abstract (web summary)

Understanding the how silicon deforms under pressure is important for several fields, including planetary science and materials design. Laser-driven shock compression experiments now confirm that shear stress generated during compression is released via a high-pressure phase transition.

## Introduction

The response of materials to ultrahigh pressures has been investigated for more than a century^1–9^, fostering our understanding of the fundamental properties of matter as well as the phenomena taking place at the interior of planets within our own solar system and beyond^10–13^. Si is one of the most abundant elements in our planet, and it has found wide-ranging application in the semiconductor industry. Because of its technological interest, Si properties have been extensively studied, both at ambient and high-pressure (HP) conditions^14–16^. However, despite decades of research, there is no consensus around the deformation mechanism that drives Si transitions at HP. Precise understanding of these transformation pathways is of interest for fields ranging from planetary science^17^ to the recovery of novel functional materials for industrial and energetic applications^18–21^.

Si exhibits a complex phase diagram, with several HP and metastable polymorphs^14^. Under static loading, upon increasing pressure Si-I transforms into metallic *β* − *tin* Si-II, *Imma* Si-XI, sh Si-V, *Cmca* Si-VI, hcp Si-VII and fcc Si-X^22–29^. Under dynamic loading, Si displays a complex response that has been a matter of debate for decades^30–32^, with a multi-wave profile emerging between 5.4 and 9.2 GPa depending upon crystal orientation and strain rate. Velocimetry experiments have identified the wave following elastic deformation with the onset of a plastic^33,34^ or inelastic^35–37^ regime, followed by a HP phase transition. Molecular dynamic (MD) simulations have suggested that, for dynamic compression of Si, HP phase transitions can occur inelastically rather than via defect-mediated plasticity. That is to say that in Si the relaxation from a purely uniaxial elastic compression to a more hydrostatic state does not happen via defect motion per se^38^, and the shear stress is released via the phase transition itself. Thus, there is not an intermediate plastic deformation regime, and the generation and motion of defects follow the phase transition rather than preceding it; in the following, we refer to this deformation regime as “transition-induced plasticity” to remark the difference with conventional defect-mediated plastic deformation. The activation of a different deformation mechanism at high strain rates could explain the different phase boundaries observed under static-^14^ and dynamic^39^ compression.

Recent in situ X-ray imaging and X-ray diffraction (XRD) of laser-driven Si compression have demonstrated that the emergence of the second wave is due to the onset of the HP phase transition, supporting the absence of intermediate plastic deformation suggested by MD^39,40^. However, these experiments could not inform the exact mechanism underlying Si structural transitions, as it requires to establish the specific orientation dependencies between the two phases. In situ XRD on shock-compressed single-crystal Si using gas gun was performed by Turneaure et al. to study the relative orientation between the ambient Si-I and the HP Si-V phase at 19 GPa^41^. However, not all the reflections could be indexed in terms of the proposed geometry, preventing an unambiguous interpretation of the experimental data. Thus, the exact deformation mechanism driving Si phase transitions under dynamic compression and the nature of the shear release mechanism (i.e., phase transition-induced- or conventional plasticity) remains elusive.

Si transformations upon decompression have been extensively studied because of the possibility to recover metastable phases at ambient conditions, such as BC8 Si-III^19,42^, Si-4H^18,43^, and other polymorphs obtained from fast decompression^44^ and confined microexplosion^45^. To date, the metastability of HP phases upon shock release has not been studied; amorphous domains have been identified in ex situ studies^46,47^, but no in situ data are available to elucidate the transformation pathway followed during decompression.

Here, we present a detailed, time-resolved XRD characterization of single-crystal Si(100) under laser-driven shock compression using an ultra-fast X-ray probe from the X-ray free electron laser (XFEL). The use of a highly monochromatic XFEL beam combined with single-crystal starting material ensures high fidelity for the analysis of single-crystal orientation and texture, which is key to investigate Si deformation mechanism. By analyzing the preferred orientation of the HP Si-V phase, we are able to determine the specific transition pathway and to provide the first direct evidence of phase transition-induced plasticity in this compression regime. Comparison with gas-gun data resolves previous controversies on the nature of Si deformation, revealing that Si exhibits a complex response to dynamic compression as shear can be released plastically or via phase transition depending on the strain rate.

## Results

**Fig. 1:**
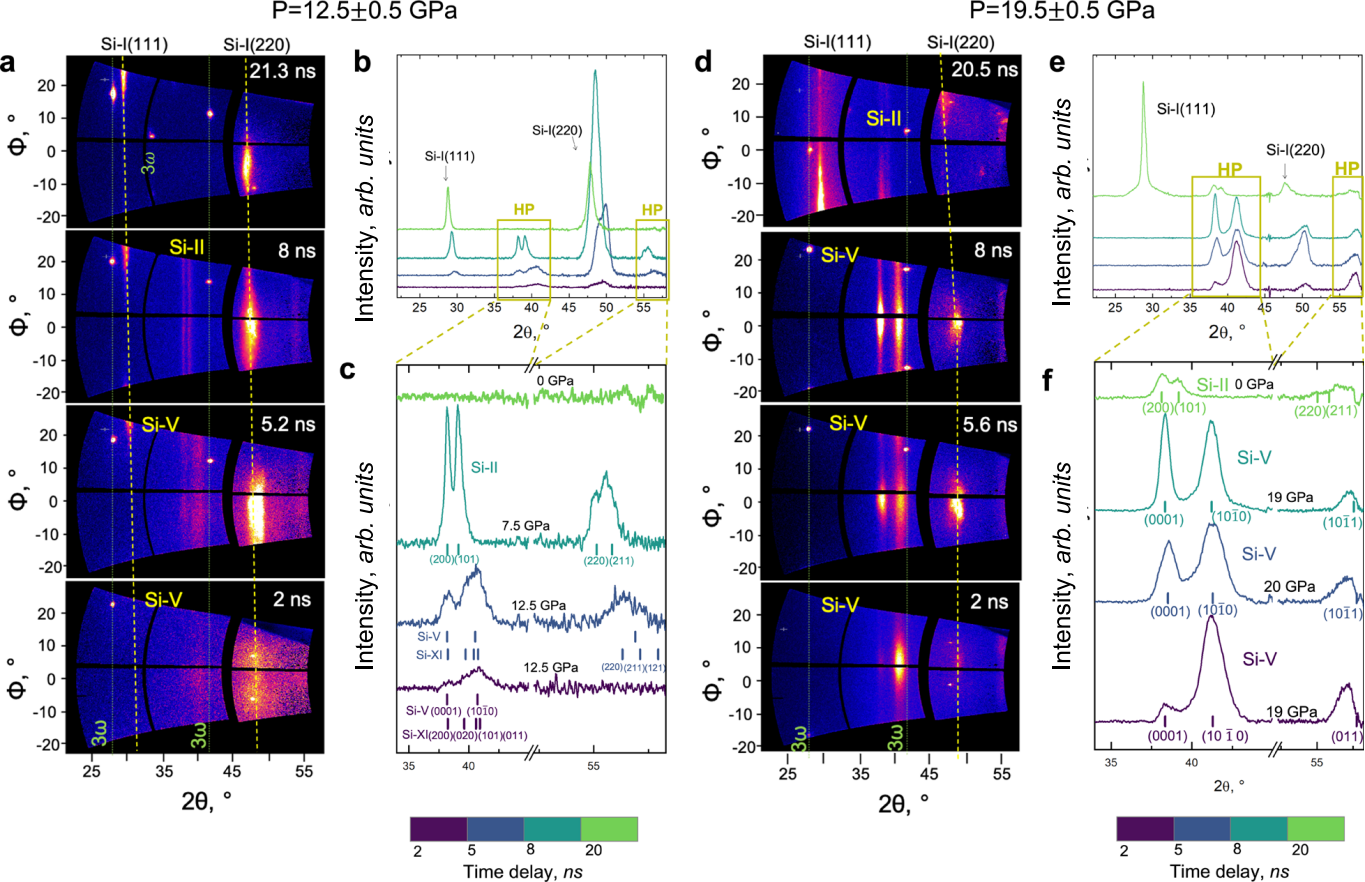
Structural evolution of Si(100) during shock compression analyzed via XRD. Data are shown for two different peak pressures: 12.5 ± 0.5 GPa (**a**–**c**) and 19.5 ± 0.5 GPa (**d**–**f**). **a**, **d** XRD data projected onto the 2*θ* − *ϕ* space acquired at different times for compression at 12.5 GPa and 19.5 GPa, respectively. The reflections from Si-I are indexed, and the yellow dashed line indicates the evolution of Si-I peaks position during compression and decompression. The green vertical lines show the position of the third-harmonic (3*ω*) reflections, which does not change over time. **b**, **c**, **e**, **f** Azimuthally integrated data are reported with different colors depending on the relative delay between the drive laser and the XFEL pulse. **b**, **e** Full XRD profile, Si-I peaks are indicated. **c**, **f** Zoomed view of the high-pressure phases structure; for each pattern, the estimated pressure is reported, and the HP phase peaks are indexed.

Experiments were conducted at the Matter in Extreme Conditions (MEC) endstation of the Linac Coherent Light Source (LCLS)^48,49^; single-crystal XRD enabled characterization of the crystal structure and microstructural changes of Si(100) under laser-driven shock compression. XRD data were acquired at time delays of 2, 5, 8, and 20-ns for two compression regimes, i.e., compression up to 12.5 GPa and 19.5 GPa (Fig. 1). Spurious reflections due to the third-harmonic (3*ω*) beam are visible in the data as well-defined single-crystal spots. These reflections were used to assess changes in the orientation of the starting material, but do not provide any information on the compression history of the sample as they come from an uncompressed region and their position does not evolve over time. The changes in the observed 3*ω* peaks form shot to shot are thus due to the slight changes in the orientation of the starting material rather than time-dependent phenomena. Upon compression, alongside the broad peaks of Si-I, new peaks corresponding to HP phases are visible (Fig. 1b, e); these peaks and those of the compressed Si-I were indexed using whole-profile fit (i.e., fitting the cell parameters to match the measured peak position). The coexistence of Si-I and HP structures is consistent with MD simulations suggesting a mixed phase region^38^ and with recent in situ XRD that reported the presence of Si-I above 19.5 GPa and well beyond the Hugoniot elastic limit^39,40^. The intensity of the XRD signal from the HP phases, on average, increases with time (more markedly at lower P), while the peak width decreases, indicating that the HP crystalline domains grow as the shock wave propagates through the material (Fig. 1c, f). At 12.5 GPa, shock compression results in a mixed HP phase comprised of both Si-XI and Si-V (Fig. 1c) in agreement with previous studies^39^. The XRD peaks observed at 2-ns and 5-ns delay fit as both Si-XI and Si-V reflections yielding consistent densities, but a precise deconvolution of the two phases is not possible due to peak superposition. At 19.5 GPa the HP structure is consistent with a pure Si-V phase, even if the asymmetry of the Si-V(10$\overline{1}$0) peaks suggests that small amounts of Si-XI may be present (Fig. 1f, see also Supplementary Information Sec. 5). Previous work has detected the onset of melting at pressures as low as 14 GPa along the Hugoniot^39^ using a transverse geometry. In our collinear geometry the sensitivity to the diffused scattering from liquid Si is reduced, which explains the lack of liquid signal at 18 GPa; the signal from liquid Si becomes detectable only at higher pressures (see Supplementary Fig. S8).

We characterized the structural transitions during both compression and decompression acquiring XRD data up to 20-ns time delay. As inferred from hydrocode simulations (Supplementary Information, Sec. 3), pressure release starts at 5.5 ns for the experiments at 12.5 GPa (43 μm-thick samples), and at 13 ns for those at 19.5 GPa (100 μm-thick samples). During release from 12.5 GPa, we observe the crystallization of Si-II at 8 ns time delay, but the phase is not preserved, and it transforms into Si-I upon complete release (Fig. 1c). The Si-II formed during decompression has a surprisingly low density, i.e., the measured atomic volume of 15.14 Å^3^/atom would correspond to a negative pressure value from extrapolation of Si-II Hugoniot curve^50^. This suggests the presence of tensile strain, most likely due to the rarefaction wave generated at the free surface of the sample after 5.5 ns. When releasing from 19.5 GPa, low-density Si-II is observed to be metastable down to ambient pressure, with a measured atomic volume of 15.16 Å^3^/atom (Fig. 1f). Despite the ultrahigh strain rate, which, according to recent DAC experiments^51^, should favor amorphization, we mainly observe Si-I recrystallization upon decompression. Most likely, the crystal–crystal phase transitions are favored by the high-temperature conditions during isentropic pressure release. It is interesting to notice that none of the metastable phases that have been synthesized in static compression experiments (e.g., Si-III^19^ or a-Si^51^) was observed. The extended metastability of metallic Si-II at ultrahigh strain rates presents opportunities for future recovery experiments that could extend the capabilities of Si HP synthesis. Indeed, the quench and recovery of HP phases from laser-driven shock compression has been demonstrated for the *ω* phase of zirconium^52^; our results suggest that this experimental approach may be applied also for the recovery of metastable metallic Si-II.

We characterize the relative orientation of the ambient- and high-pressure phases to gain new insight into the atomistic deformation mechanisms that drive Si phase transitions. Indeed, a given deformation mechanism will result in a specific orientation relationship, which can be identified in dynamic compression experiments because shock-compressed materials, being inertially confined, typically exhibit a lower mosaicity compared to static loading. In this study, the use of a highly monochromatic XFEL beam combined with single-crystal starting material ensures an ultra-fast probe for time-resolved study and high fidelity for single-crystal characterization. Upon compression, the XRD signal intensity is not uniform along the Debye–Scherrer rings, confirming the low mosaic spread and suggesting that the HP crystallites grow along preferred directions (Fig. 1a, d). In particular, Si-I(220), Si-V(0001), and Si-V(10$\overline{1}$0) reflections are centered around the same *ϕ* angle (Fig. 1d). The observed relative orientation was reproducible and obtained under different compression regimes, suggesting that it is intrinsic to the transition mechanism (Supplementary Information, Sec. 4). If dynamic compression resulted in the growth of single-crystal HP phases, Si-V(0001) and Si-V(10$\overline{1}$0) reflections would appear at 90^∘^ in reciprocal space, as they correspond to perpendicular planes. Thus, Si-V reflections are from two distinct crystalline domains with highly reproducible relative orientation. With the new insights from our XFEL-based experimental approach, we have analyzed several potential orientation relationships, including those proposed in ref. 41, to explain the highly preferred orientation of Si HP phases under dynamic compression.

**Fig. 2:**
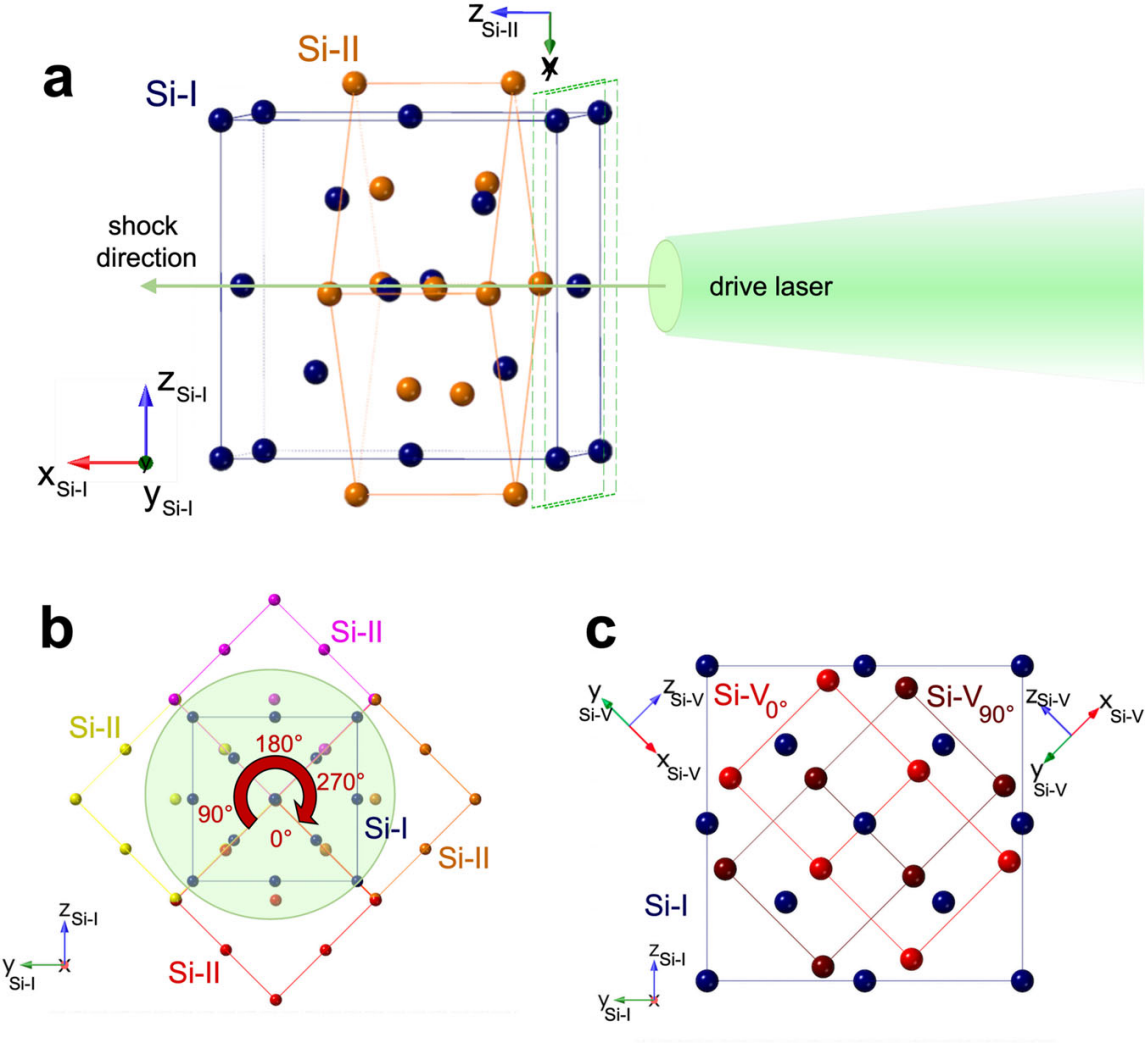
Schematic view of the orientation relationship. **a** Preferential orientation for the nucleation of Si-II (orange) from shock-compressed Si-I (blue): [001]_*II*_//[100]_*I*_, i.e,. the compression direction. **b** Perpendicularly to the shock: Si-I (blue) and four equivalent Si-II orientations, each of which is indicated with a different color. **c** Orientation relationship between the ambient pressure Si-I (blue) and the HP Si-V phase (red), for which two non-equivalent domains, Si-${V}_{{0}^{\circ }}$ and Si-${V}_{9{0}^{\circ }}$ are indicated.

**Fig. 3:**
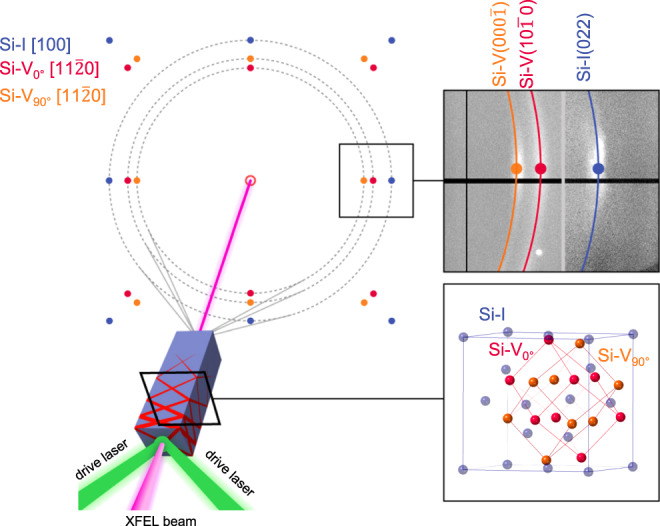
Schematic view of our experimental configuration and deformation mechanism. The calculated XRD pattern fits our experimental data (right upper panel). The position of the single-crystal diffraction spots is calculated using the proposed orientation relationship between Si-I and Si-V, while the circles and lines serve as a guide for the eye and a reference for the correspondent Debye–Scherrer rings.

We tested the specific orientation relationship dictated by transition-induced plasticity in Si(100), i.e., the shear stress is released via the phase transition as proposed by MD simulations^38^. It is worth noting that the transition geometry proposed by MD refers to the *Imma* Si-XI phase, rather than the hexagonal Si-V phase observed in our data. However, because these phases are linked by a displacitive transition along [001]_*XI*_, once the relative orientation of Si-XI and Si-V basis vectors is taken into account the expected orientation of the Si-V phase can be easily deducted from the MD model. The initial elastic compression occurs uniaxially along [100]_*I*_, i.e., the [100] direction of the Si-I cubic crystal, generating shear stress in the sample. In order for the system to reach the hydrostat inelastically (i.e., without generation of crystalline defects), as part of the sample changes structure to Si-II, the remaining Si-I must experience compressive stress perpendicularly to the shock in the (011)_*I*_ plane. For the Si-I → Si-II transition, this is possible if the tetragonal HP phase crystallizes along a specific orientation: [001]_*II*_//[100]_*I*_, i.e., the *c* axis of the HP phase, with shorter inter-atomic distances, is parallel to compression axis (Fig. 2a). Perpendicularly to the shock, in the (011)_*I*_ plane, Si-II inter-atomic distances are higher, as shown in Fig. 2b, where the basis vectors of Si-II are rotated through an angle of 45^∘^ with respect to the cubic Si-I cell. As a result, if the transition happens with this specific orientation relationship, the relaxation toward the hydrostat (i.e., release of shear stress) is caused by the transition to the highly-oriented HP Si-II phase, and it does not require the generation or motion of crystallographic defects. It is worth noting that this Si-I - Si-II orientation relationship has also been investigated by ab initio calculations, and found in good agreement with static compression experiments^53^. Because of the fourfold symmetry of the tetragonal phase, there are 4 equivalent crystalline orientations for Si-II in the (011)_*I*_ plane (Fig. 2b). As Si-II transforms into *Imma* Si-XI and hexagonal Si-V via continuous deformation, the crystal structure is distorted, and the fourfold degeneracy is lost (*a*_*II*_ = *b*_*II*_ → *a*_*XI*_ ≠ *b*_*XI*_). The transition to Si-V ultimately results in the formation of crystalline domains with two non-equivalent orientations, as shown in Fig. 2c: (i) Si-${V}_{{0}^{\circ }}$, with [110]_*V*_//[100]_*I*_ and ${[001]}_{V}//{[0\overline{1}1]}_{I}$; (ii) Si-${V}_{9{0}^{\circ }}$, with [110]_*V*_//[100]_*I*_ and [001]_*V*_//[011]_*I*_. The single-crystal XRD pattern calculated using this orientation relationship fits our experimental data well (Fig. 3), while the other geometries we tested could not explain our results. We are thus able to unambiguously interpret our XRD data in terms of a specific deformation mechanism. The sequence of HP phase transitions under uniaxial shock compression results in two non-equivalent Si-V orientations at an angle of 90^∘^, which explains the observation of both Si-V(0001) and Si-V(10$\overline{1}$0) around the same *ϕ*.

**Fig. 4:**
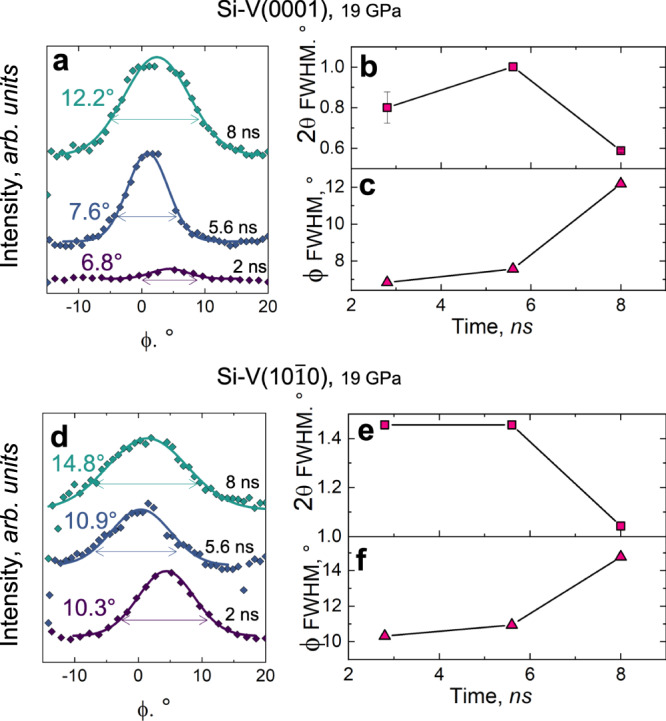
Microstructure analysis. **a**, **d** *ϕ* lineouts for Si-V(0001) and Si-V(10$\overline{1}$0) peaks at different times. Experimental data are reported and overlapped with peak fitting results (continuous line and FWHM values form fit). **b**, **c**, **e**, **f** Time evolution of peak width along 2*θ* and *ϕ* for both analyzed peaks. The error bars show the uncerainty over the FWHM value as estimated by the fit of the experimental data.

We analyzed the XRD peaks‘ shape to gain insight on the microstructure of shock-compressed Si, e.g*.*, changes in mosaicity, grain size, crystallographic defects. The Si-V(0001) and Si-V(10$\overline{1}$0) reflections observed around the same *ϕ* value necessarily come from two distinct crystal domains, which prevents analysis of the microstructure via whole-profile refinement (e.g., Le Bail); therefore, we used single-peak fitting projecting the XRD spots along both the 2*θ* and *ϕ* axis (Fig. 4, see also Supplementary Information, Sect. 5). The Full Width at Half Maximum (FWHM) along *ϕ* gives an estimate of the disorder of and within the crystalline domains, i.e., a combination of the mosaicity (the angular spread of the orientations of the crystallites^54^) and rotation of the lattice planes^55^ (Fig. 4a, d). The *ϕ* lineouts of Si-V peaks at 19.5 GPa show that the peak width along *ϕ* increases with time (Fig. 4c, f) in conjunction with the growth of the HP crystallites, i.e., with the decrease of the 2*θ* FWHM (Fig. 4b, e). At 12.5 GPa we observe a similar trend, and the mosaicity increase results in a powder-like texture when Si-II is formed during decompression (Fig. 1a). In this regime, the XRD peaks are generally broader and less intense, preventing a quantitative analysis of mosaicity. XRD analysis suggests thus nucleation of highly-oriented HP crystalline domains, with a gradual loss of preferred orientation as the crystalline domains grow. These trends are consistent with the model of HP nano-crystalline domains originating from shear bands along the specific crystalline direction, e.g., the Si-I[111] suggested by MD^38^. It is worth noting that previous ex situ characterization of shock-compressed Si(100) also showed that amorphous bands originate along {111} slip planes, and, as they grow, they tend to deviate from this orientation and align with the direction of maximal shear (i.e., 35.3^∘^ with shock direction in Si(100))^46,47^.

**Fig. 5:**
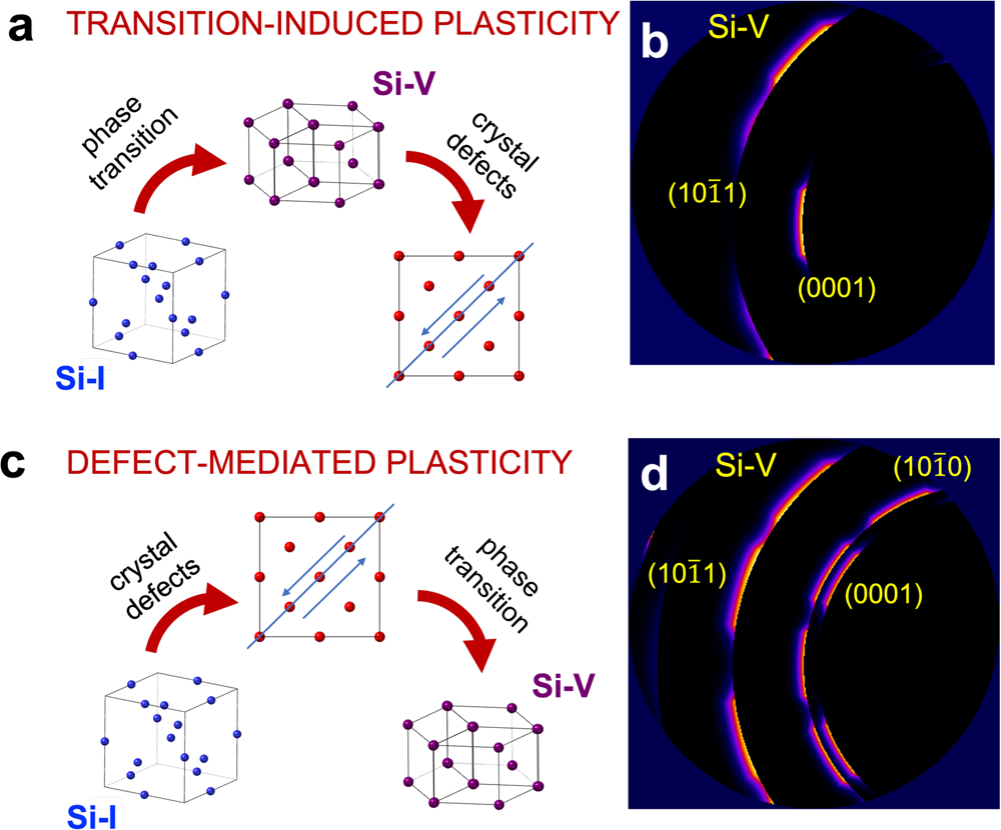
Analysis of defect-mediated and transition-induced plasticity and their XRD signature. **a**, **c** Schematic view of the two deformation mechanisms. **b**, **d** Corresponding XRD signature from forward calculations using the orientation relationship proposed in this work and a pink beam (Supplementary Information, Sec. 6^50^).

Comparison with previous gas-gun experiments provides additional insights into Si deformation under dynamic compression. The differences between our results and previous experiments in ref. 41 (who suggested different orientation relationships that could not explain all the peaks in gas-gun data) could be due to the specifics of the diagnostics, e.g., the use of a pink beam, or by the lower strain rate of gas-gun compression, that may activate different deformation mechanisms^37^. In order to perform a direct comparison with the data by Turneaure et al., we performed forward XRD calculations using the orientation relationship proposed in this work and the experimental parameters of previous gas-gun studies (for details, see Supplementary Information, Sect. 6). XRD patterns were simulated for both transition-induced and defect-mediated plasticity. As shown by the schematics in Fig. 5a, the former is characterized by shear release via a phase transition, followed by the generation of defects and texture evolution; the growth of the HP phase happens only along a specific orientation, resulting in highly localized XRD signal (Fig. 5b). Instead, when the sample deforms plastically, the system relaxes toward the hydrostat via generation and motion of defects before the phase transitions take place (Fig. 5c). After plastic shear release, the compression is not uniaxial and the HP phase can crystallize isotropically, which results in the additional visible reflections shown in Fig. 5d, where the XRD signal was calculated including all the equivalent variants of the given orientation relationship. Thus, our calculations demonstrate that these deformation regimes result in different crystalline geometries, with clearly distinguishable XRD signatures, which explains the differences between our results and previous gas-gun studies^41^. At lower strain rates, like in gas-gun experiments, shock-compression results in defect-mediated plasticity, while the shear release via phase transition characteristic of laser-ablation experiments induces a strongly preferred orientation for the growth of the HP phases. With the new insight provided by our time-resolved single-crystal analysis, we can explain the discrepancies in previous studies on shock-compressed Si, resolving a long-standing debate.

In this study, we propose the first unified model for Si deformation under shock compression, which has been a matter of vigorous debate for decades. Time-resolved XRD is used to characterize, in situ, the phase transitions of single-crystal Si(100) under laser-driven shock compression. With this experimental approach, we provide the first unambiguous, atomistic picture of the deformation mechanism driving phase transitions under laser-driven compression. We observe crystallization of the Si-V HP phase above 12 GPa, and provide evidence of extended metastability of metallic Si-II upon release. The use of a monochromatic XFEL probe with sub-picosecond temporal resolution allows us to determine the orientation relationship between the Si-V and Si-I phases, connecting it with the specific deformation mechanism and confirming the shear release via phase transition predicted by MD simulations^38^. Analysis of the microstructural evolution demonstrates that, at ultrahigh strain rates, the HP Si phases form along specific directions with a loss of preferred orientation and generation of defects during crystal growth. Crucially, a comparison with previous gas-gun experiments demonstrates that at different strain rates, the deformation mechanism and the geometry of the phase transition can be substantially different, resolving the ongoing debate around shock-compressed Si.

## Methods

Single-crystal, double-side parallel-polished platelets of Si(100) (either 43-μm or 100-μm thick) were mounted such that the starting orientation with respect to the probe direction and the shock propagation direction could be tracked. The LCLS delivered 60-fs duration quasi-monochromatic X-ray pulses of energy 7.952 keV (Δ*E* = 15-40 eV^56^, Δ*E*/*E* = 0.2−0.5%). The XRD signal was recorded on two Cornell-SLAC Pixel Array Detectors covering range of scattering angle 20^∘^ < 2*θ* < 70^∘^ and azimuth −25^∘^ < *ϕ* < 25^∘^^49^. In this experimental geometry, no XRD signal from the single-crystal starting material is observed for the monochromatic 7.952 keV X-ray probe; spots from Si-I are visible only from crystallites in appropriate orientation and upon compression, when mosaicity and strain increase. However, additional spots due to the parasitic third-harmonic beam are always visible and allow us to evaluate changes in the orientation of the starting material. Shock compression was achieved by direct irradiation of the sample by a 527-nm laser with a quasi-flattop pulse profile of 10-ns duration^57^ with 1 × 10^12^ W/cm^2^ intensity. We collected data at time delays up to 20 ns, which enabled characterization of the transformations taking place during both compression and release, i.e., after the shock wave has traversed the sample. During compression, the pressure was estimated from the experimental density obtained from XRD and the multi-phase equation of the state of Si^50^. Indeed, the only detectable XRD signal comes from the compressed region, which consists of a uniform HP state. Velocimetry data obtained using the velocimetry interferometry system for any reflector (VISAR) diagnostic and hydrocode simulations were used to confirm pressure and strain-rate estimation (Supplementary Information Sec. 2–3). After the shock reaches the rear surface of the sample, a release wave back-propagates through the sample; both the isentropic release pathways, i.e., off-Hugoniot state, and the inhomogeneity of the pressure state in the sample prevent the use of Si EOS to estimate pressure. For these reasons, after breakout the pressure evolution was inferred from hydrocode simulations. By varying the time delay between the drive laser and the XFEL beam, time-resolved XRD data were collected at different pressures. For more details, see Supplementary Information Sec. 1.

## Supplementary information

Supplementary Information: Atomistic deformation mechanism of silicon under laser-driven shock compression

Editorial Assessment Report

## Data Availability

The data that support the findings of this study are available from the corresponding author upon reasonable request.
