# Supplementary material for: Atomistic deformation mechanism of silicon under laser-driven shock compression
Source: Nat Commun. 2022 Sep 21;13:5535. doi: 10.1038/s41467-022-33220-0 (PMC9492784; doi:10.1038/s41467-022-33220-0)
Supplement: Supplementary file 1 — Supplementary Information: Atomistic deformation mechanism of silicon under laser-driven shock compression [file 41467_2022_33220_MOESM1_ESM.pdf]

Supplementary Information: Atomistic deformation mechanism of silicon under laser-driven shock compression

S. Pandolfi[1], S. Brennan Brown[1], P.G. Stubley[2], A. Higginbotham[3], C. A. Bolme[4], H. J. Lee[1], B. Nagler[1], E. Galtier[1], R. Sandberg[4*], W. Yang[5], W. L. Mao[6], J.S. Wark[2], A.E. Gleason[1]

1. SLAC National Accelerator Laboratory, 2575 Sand Hill Rd., Menlo Park, CA 94025, USA.
2. Department of Physics, Clarendon Laboratory, University of Oxford, Parks Road, Oxford, OX1 3PU, UK
3. Department of Physics, University of York, Heslington York, YO10 5DD, U.K.
4. Los Alamos National Laboratory, Los Alamos, New Mexico 87545, USA
5. Center for High Pressure Science and Technology Advanced Research, HPSTAR, Shanghai, China
6. Geological Sciences, Stanford University, 367 Panama St., Stanford CA 94305, USA.
* Now at: Department of Physics and Astronomy, Brigham Young University, Provo, Utah 846002, USA

1. Detailed experimental method

Using the Matter in Extreme Conditions instrument at the LCLS [1,2], quasi-monochromatic (dE/E=0.2–0.5%), fully transverse coherent, 7.952(30) keV X-ray pulses of 60-fs duration with an average of 10^{12} photons per pulse were incident over a 75-µm diameter spot on the target package (Figure S1). An X-ray-only shot was collected before each drive shot as a reference. The 75-µm XFEL beam spot did not produce any observable X-ray damage to the target. Using different phase plates on the optical drive laser, a 200- or 400-µm diameter flat-top laser spot was used to achieve focal spot intensity of 1×10^{12} W/cm^2. The angle between the drive laser arms and XFEL probe is 6° (Figure S1).

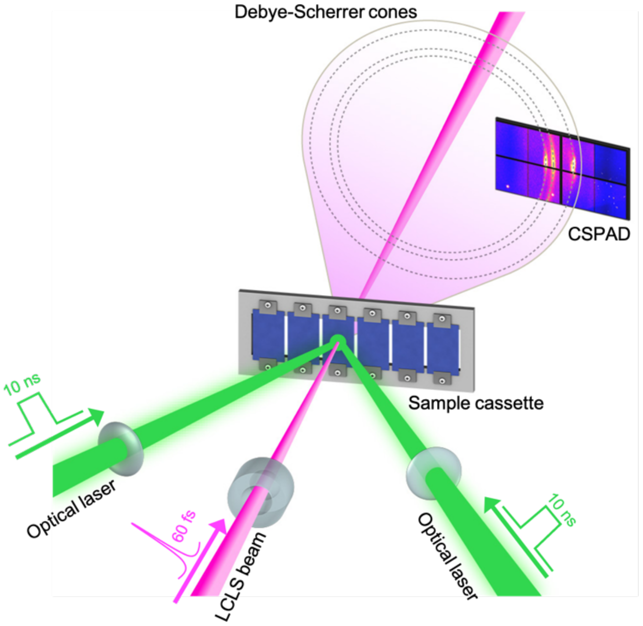

Figure S1. Schematic view of the experimental setup.

An ablation-driven compression wave was launched parallel to the sample normal over a 10-ns quasi-square pulse profile from a frequency-doubled Nd:Glass laser system (λ=527 nm). The optical laser and X-ray beam were spatially overlapped and operated in single-shot mode. The absolute time zero corresponds to overlap of their leading edges. For each shot, a time delay was selected for the XFEL pulse relative to the optical laser pulse with a jitter of 0.3–0.5 ns. This delay time was verified by oscilloscope traces captured for each shot. The pump–probe delay scans at several nanosecond intervals enabled collection of a time series of XRD patterns in transmission geometry. XRD patterns were captured by CSPADs constructed of individual application-specific integrated circuits [3]. Additional spots from the XFEL 3rd harmonic beam (3ω, with energy 23.86 keV) are

visible. The spots are most likely coming from the uncompressed Si-I surrounding the drive area, as the 3ω beam is not focused on the sample and the spots position does not change with pressure nor before and after shock. Since the optical path of the 3ω beam is not well characterized, quantitative analysis of these reflections is not possible. However, the 3ω reflections provide qualitative information on the starting material's orientation. For example, the 3ω spots in Fig.1(d) in the main text shows how the changes in the peaks position between the 8-ns and 20-ns time delay are due to a change in the starting material rather than to a relative rotation of the crystallites.

2. VISAR analysis

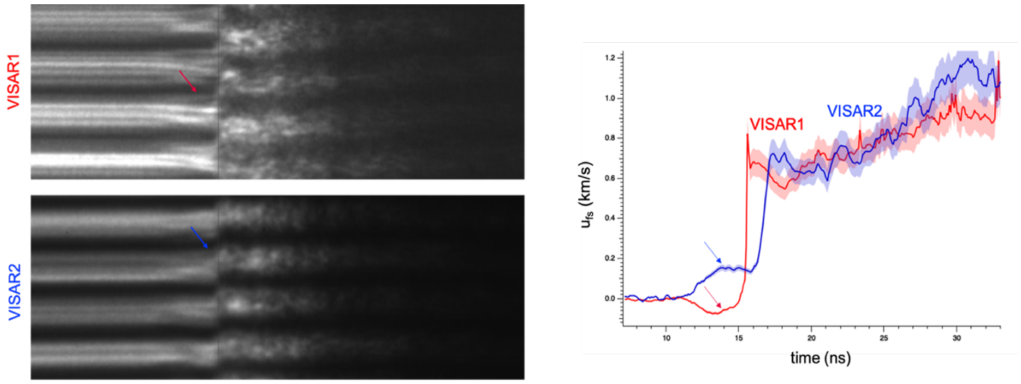

Figure S2. Left: representative VISAR images for shock-compressed Si(100). Right: VISAR1 and VISAR2 traces.

Velocimetry data was obtained by analyzing the line VISAR [4] interferograms. The velocity of the free surface, u_{FS}, was measured using two VISAR systems with different velocity sensitivity. VISAR1 used a 50 mm etalon of fused silica, resulting in a velocity per fringe (VPF) relation of 0.996 km/s, and VISAR 2 used a 15 mm etalon, with a VPF of 3.320 km/s; both VISAR systems used a 50 ns streak window to measure u_{FS} as a function of time. LLNL's AnalyzeVISAR code was used to analyze the data, and the measured free surface velocity was determined when both VISARs reached agreement (Figure S2). The central fringes from the line VISAR were spatially averaged to provide a velocity profile to be used as a boundary condition for forward hydrodynamic simulations (Sect.3). Representative VISAR traces for different sample thicknesses and peak pressures are shown in Figure S3. The quality of the data is not sufficient to extrapolate the entire compression history for each sample. However, the fringe shift when the shock reaches the sample's free surface can be quantitatively analyzed and it is consistent with the expected elastic response for Si.

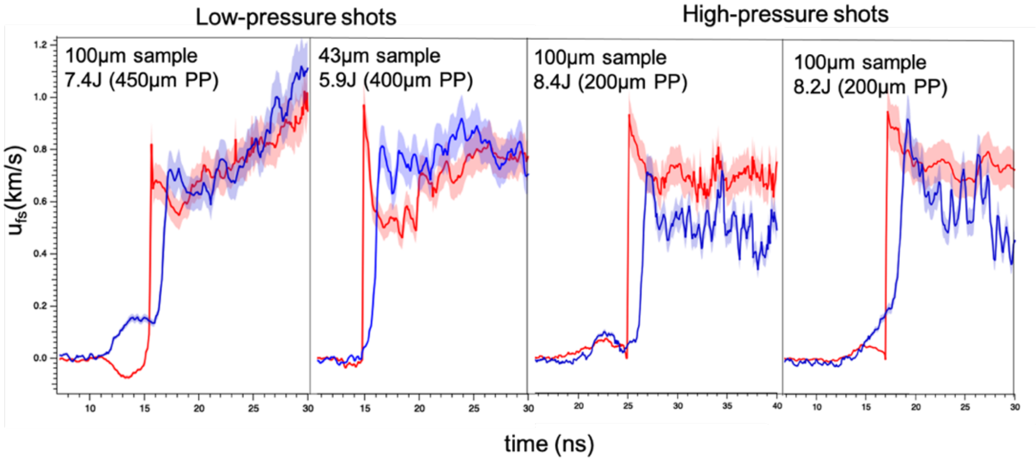

Figure S3. Example of free surface velocity traces for both VISAR1 and VISAR2. Samples compressed up to different peak pressures and of different thicknesses have been analyzed. It is worth noting that the time axis of the streak camera can be arbitrarily offset, and thus the breakout time as measured by VISAR does not correspond with the transit time of the shock wave through the sample.

3. Hydrocode simulation

We performed 1-dimensional (1D) hydrodynamic simulation using the HELIOS Collisional-Radiative code [5], to achieve information on the pressure spatial distribution throughout the Si wafers. The target in the simulation is consistent with the experimental target, i.e., either 43 µm- or 100 µm-thick Si. The quotidian equation of state of silicon is calculated using the PROPACEOS software [5]. The experimental drive profile, recorded for every shot, is used as input for the laser driver in the simulation. The drive intensity is adjusted in order to correct for the energy deposition and transfer in a 1D model. The intensity multiplier coefficient is determined by comparing the simulated pressure to the pressure calculated from the XRD data using the Si equation of state [6], and comparing the calculated particle velocity with the velocimetry data from VISAR.
The results of hydrocodes simulations for different sample thicknesses explain the differences between the measured pressure values at 8 ns time delay for the 19.5- and the 12.5-GPa time series (Figure 1 of the main text). For the experiments at 12.5 GPa, thinner 43 µm-thick samples were used; in this case, the compression wave reaches the rear surface of the sample after 5.5 ns (Figure S4(a)), launching a back-travelling rarefaction wave due to the impedance mismatch with vacuum. Thus, at 8 ns time delay, the XRD signal in 43 µm-thick samples comes from portions of the sample subjected to either a compressive or a tensile (i.e., negative pressure) stress, which can explain the observation of metastable low-density Si-II phase. For the experiments at 19.5 GPa, the use of thicker 100 µm samples results in longer transit times, so that at 8 ns time delay part of the sample is still uncompressed (thus, being a single crystal, does not contribute to the XRD signal), and the compressed portion of the sample is in a homogenous high-pressure state (Figure S4(b)).

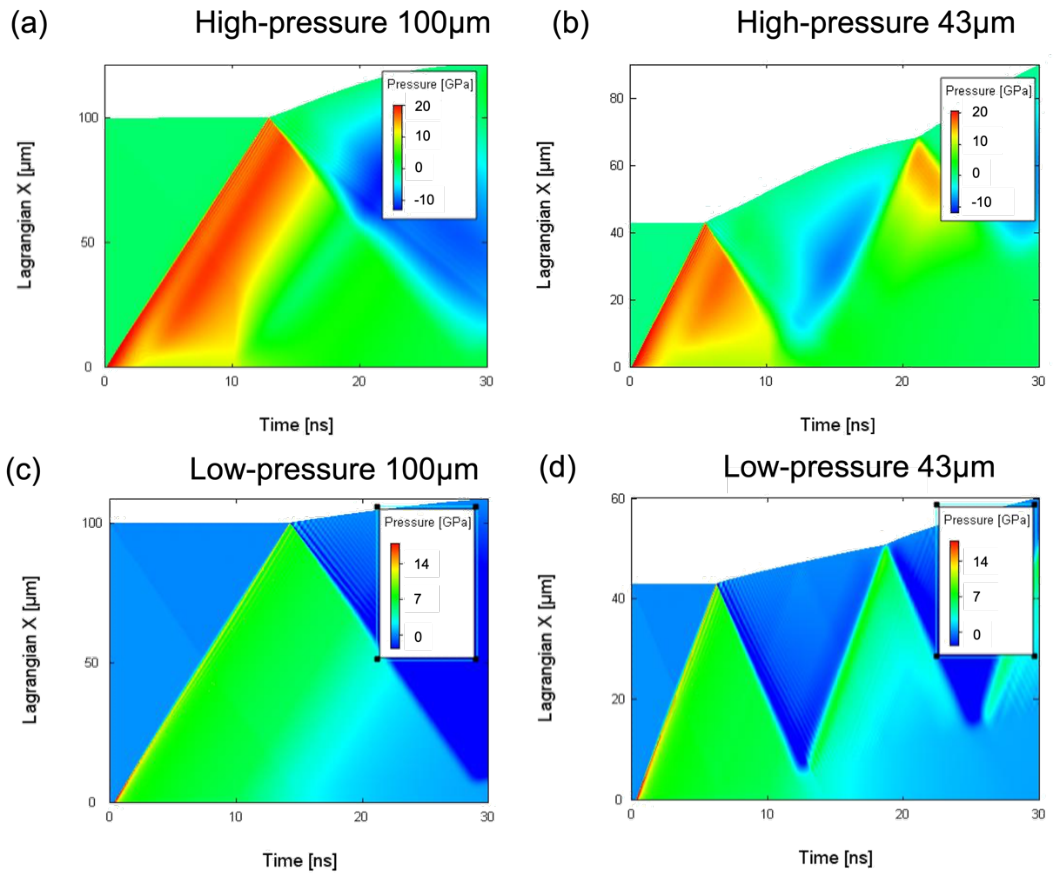

Figure S4. Hydrocode simulation for sample compressed at 18-20 GPa: (a) 100µm-thick (b) 43µm-thick. Hydrocode simulation for sample compressed at 10-12 GPa: (c) 100µm-thick (d) 43µm-thick.

4. XRD Datasets

In this section, two additional datasets are presented, showing time resolved XRD at peak pressure close to the ones analyzed in the main text: 11 GPa (Figure S5) and 17 GPa (Figure S6). The observed trends are consistent with the data reported in Figure 1 in the main text, demonstrating the reproducibility of our results.
After compression up to 11 GPa, metastable Si-II is seen at 9 ns time delay during release; in this case, minimal traces of the HP phase are still visible at 0 GPa (Figure S5). During compression up to 17 GPa of 100µm-thick samples, at 9 ns time delay pressure release has not yet started, and the observed HP phase is compatible with hexagonal Si-V (Figure S6). The extent of the XRD peaks along the Φ axis, i.e. the vertical axis of the bidimensional projected patterns, shows that the nucleation of HP phases takes place along preferential directions, and that the microstructural disorder (either mosaicity or plane distortion) increases with time, up to a complete loss of preferential orientation (i.e., a powder-like texture) when Si-II is formed during release (Figure S5).

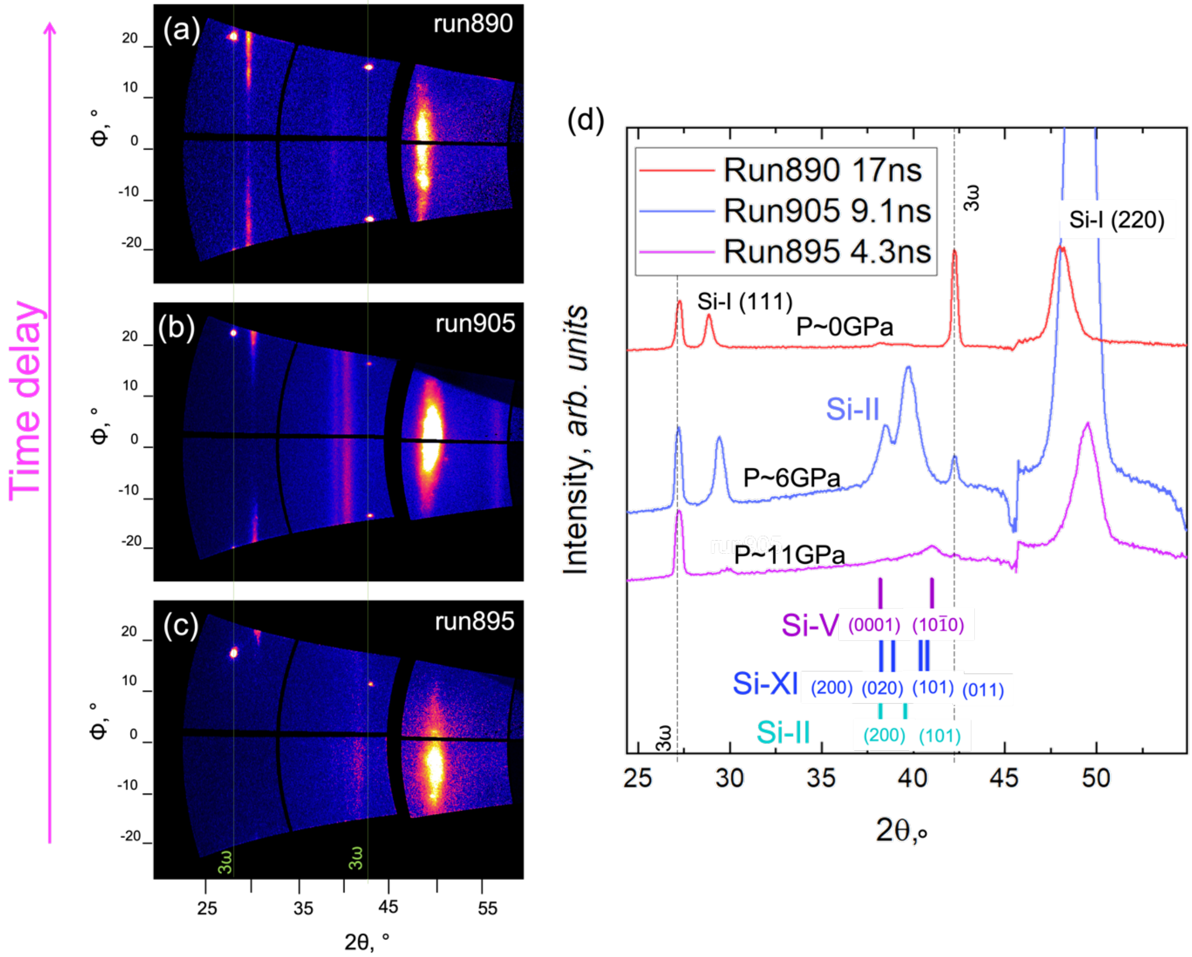

Figure S5. Time-resolved XRD for shock-compression up to 11 GPa: (a-c) projected $2\theta - \Phi$ patterns, (d) azimuthally integrated XRD patterns. No background subtraction or 3ω filtering has been applied to the data. With the use of 43 µm-thick samples, pressure release starts at 5.5 ns time delay, as a rarefaction wave propagates back through the sample form the rear surface. Vertical dashed lines indicate the reflections from the 3ω beam; the ideal peak position for each HP phase is also reported in (d).

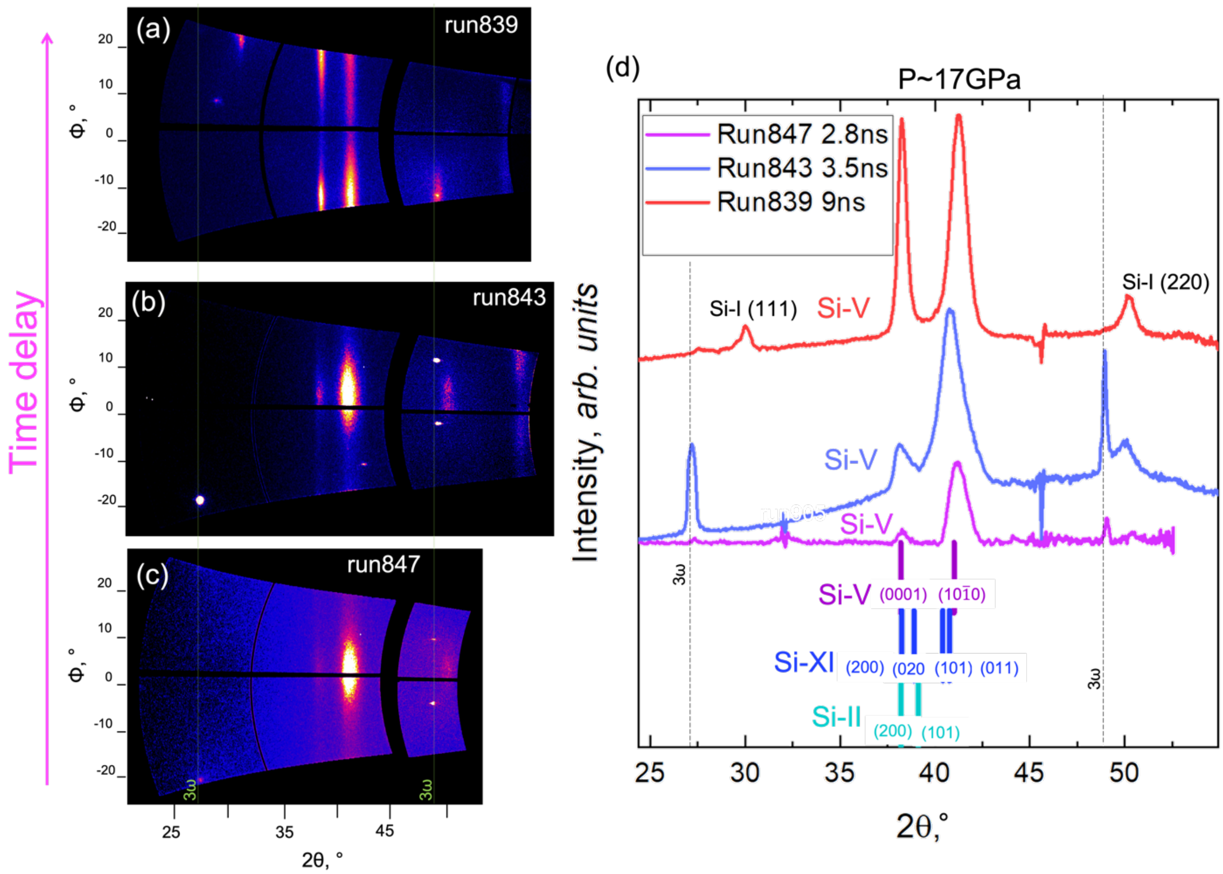

Figure S6. Time-resolved XRD for shock-compression up to 17 GPa: (a-c) projected $2\theta - \Phi$ patterns, (d) azimuthally integrated XRD patterns. No background subtraction or 3ω filtering has been applied to the data. The intensity of the Si-V XRD peaks increases over time (i.e., as the shock waves propagates through the sample). Vertical dashed lines indicate the reflections from the 3ω beam; the ideal peak position for each HP phase is also reported in (d).

5. Phase Identification

In this Section, the results from our XRD fitting procedure and the criteria used for phase identification are explained in detail. According to our proposed transition mechanism, the XRD peaks observed in our experimental data come from two different crystalline domain of the HP phases tilted of 90°. For this reason, we did not use Le Bail or equivalent fitting methods, as no unique set of size and strain parameters could describe the microstructure of both domains and fit the shape of both peaks. We have thus fit the 2θ peaks one by one to obtain peak center and FWHM, as shown in Figure S7. The peaks positions were then used to obtain the corresponding cell parameters for the different HP phases. The results are reported in Table S1. Given the limited number of XRD peaks that could be reliably deconvoluted and fitted, we have a limited number of constraints when fitting the cell parameters, which prevents estimation the uncertainty on the values.

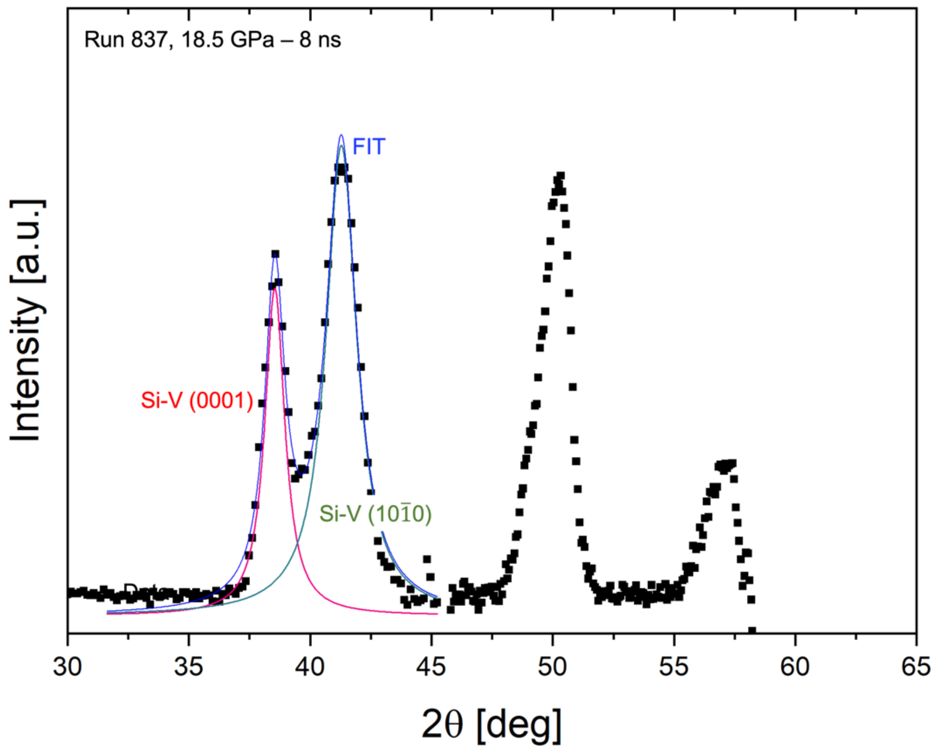

Figure S7. Representative XRD analysis showing the fit results for data acquired at 18.5 GPa and 4 ns time delay. The peaks are fitted singularly to measure the position and FWHM.

The crystal structure of Si-II, Si-XI and Si-V HP phases are closely related: the orthorhombic *Imma* structure of Si-XI can be obtained by lifting the *a*=*b* condition of the tetragonal Si-II symmetry, and it has been suggested to define a continuous deformation pathway towards the hexagonal Si-V [7]. Besides the *b/a* ratio, another distinguishing feature is the value of the *c/a* ratio, which has been shown to evolve with pressure from 0.55 for Si-II, to 0.534 for Si-V [7–9]. We fit our experimental data using the structural models of both Si-XI and Si-V, which have been observed above 10 GPa [10]. The results are shown in Table S1, where we analyze the relative atomic volumes, i.e., V/V_0. Our results demonstrate that at 12 GPa, fit using both Si-XI and Si-V crystal structures yields consistent densities (and corresponding pressures), suggesting that coexistence of the two phases is possible. Furthermore, the signal intensity at this pressure is quite low, and the XRD peaks broad, such that precise deconvolution of different phases is not possible. On the contrary, for the data collected at 20 GPa, the densities obtained using either Si-XI or Si-V are not consistent and correspond to different pressure values according to Si equation of state [6] (shown in parentheses in Table S1), thus ruling out the presence of a mixed phase.

P	Time	Fit Si-XI			Fit Si-V		*c/a*		V/V_0		Phase
GPa	ns	a_{Si-XI} [Å]	b_{Si-XI} [Å]	c_{Si-XI} [Å]	a_{Si-V} [Å]	c_{Si-V} [Å]	c/a_{Si-XI}	c/a_{Si-V}	V/V_{0Si-XI} (P)	V/V_{0Si-V} (P)	
12	2.0	4.76	4.60	2.55	2.59	2.38	0.54	0.53	0.70 (11.4)	0.69 (12.7)	--
12	5.2	4.76	4.59	2.56	2.60	2.38	0.54	0.53	0.70 (11.4)	0.70 (10.5)	--
20	2.8	4.72	4.62	2.56	2.56	2.37	0.54	0.53	0.70 (11.4)	0.67 (18.4)	Si-V
20	5.6	4.70	4.60	2.55	2.55	2.36	0.54	0.53	0.69 (14.7)	0.67 (18.4)	Si-V
20	8.0	4.74	4.58	2.54	2.56	2.37	0.54	0.53	0.69 (14.7)	0.67 (18.4)	Si-V

Table S1. Results from XRD patterns fit using different HP phases. The pressures corresponding to the measured V/V_0 for both Si-XI and Si-V are reported in parentheses and the values are in GPa.

Figure S8 shows XRD patterns acquired at 4 ns time delay (thus during compression) at increasing pressure. We notice that, at 13 GPa, a doublet is visible around 38° (Figure S8(c)), while at 15 GPa the second peak is not clearly visible (Figure S8(b)). The presence of the doublet is a distinctive feature of the *Imma* structure of Si-XI: as $a_{II}=b_{II} \rightarrow a_{XI} \neq b_{XI}$, the (200) and (020) reflections of SI-XI become distinguishable. Furthermore, according to our proposed deformation mechanism, two domains rotated of 90° should be formed upon compression, which would make the Si-XI(200)$_{0°}$ and the Si-XI(020)$_{90°}$ reflection to appear at the same ϕ, which is consistent with our data (Figure S8(c)). At 15 GPa, the intensity ratio of the XRD peaks has changed, and the doublet is not clearly visible, suggesting that the predominant phase at this pressure is Si-V rather than Si-XI. Despite the presence of small amounts of Si-XI phase cannot be ruled out given the broad and asymmetric XRD peaks at 15 GPa, the high quality of our data and the peculiar geometry of Si deformation mechanism ensure that the distinctive features of Si-XI and Si-V phases are distinguishable in our XRD patterns.

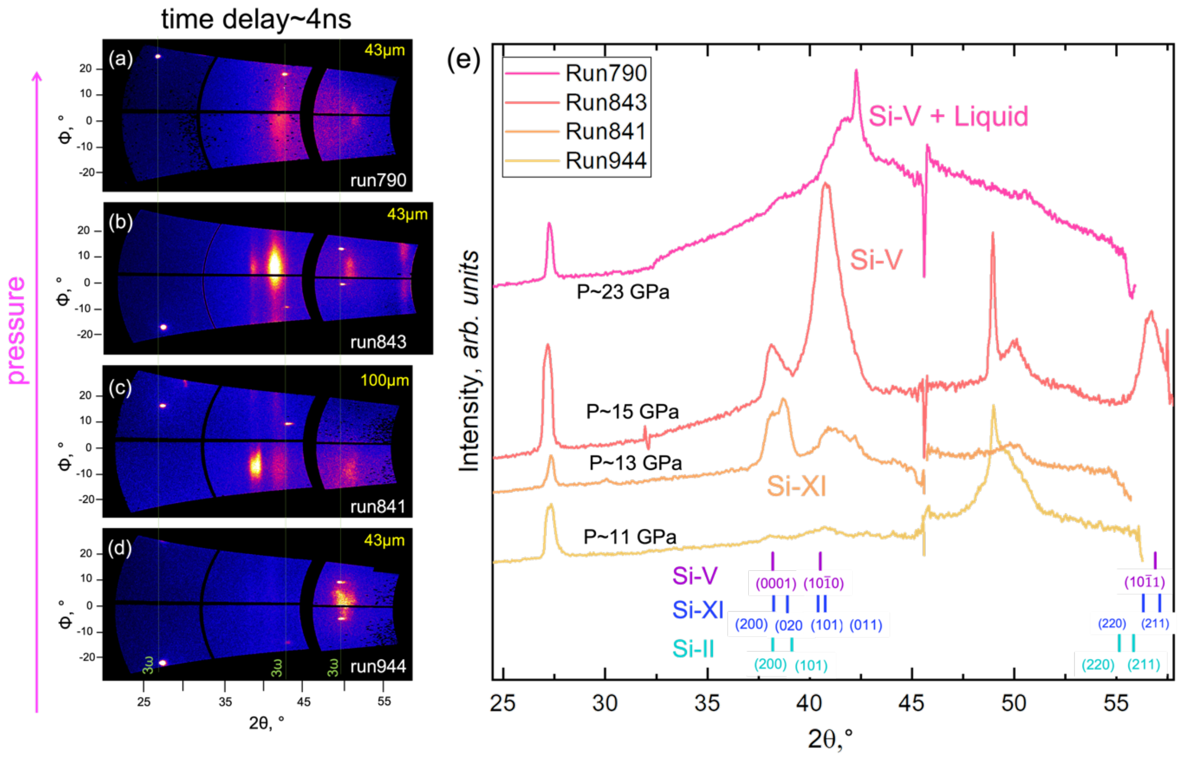

Figure S8. XRD data acquired at 4 ns time delay for increasing laser energy: (a-d) projected $2\theta - \Phi$ patterns, (e) azimuthally integrated XRD patterns. No background subtraction or 3ω filtering has been applied to the data. The different HP phases identified at varying pressure are indicated for each pattern; the ideal peak position for each HP phase is also reported in (e).

6. XRD simulations

In order to simulate the expected signal from our proposed orientation relationship in the experimental geometry of Turneaure et al. [11], a code was developed to predict the diffraction from textured material in a pink beam experiment. This code conceptually follows the work from Turneaure, but with some modifications and simplifications for efficiency. A 750x750 pixel detector is defined in a geometry consistent with Turneaure's, and the direction of $\mathbf{G}_{pix}=\mathbf{k}-\mathbf{k}_0$ (calculated for unit $\mathbf{k}$ and $\mathbf{k}_0$) for scattering to each pixel is determined. For each variant of the orientation relationship (OR), the reciprocal lattice for Si-V is calculated. The sample normal is assumed to be tilted at 6° to the incoming x-ray beam. The reciprocal lattice vector $\mathbf{G}$ is calculated for all allowed

(*hkl*), and for each pixel the photon energy required for diffraction with scattering vector of length |**G**| to each pixel is calculated. An intensity is added to each pixel for every (*hkl*) based on three factors. The intensity is initially set by pink beam spectrum intensity at the required photon energy and is then further modified by the atomic form factor. The main difference with our work is that, in a pink beam configuration, the x-ray probe has the sensibly larger bandwidth, which results in a softening of the geometric constraint to satisfy Bragg law. This implies that, in the pink beam configuration, the XRD signal from domains with up to several degree of misalignment would be equally visible, reducing the sensitivity of the measurements with respect to a specific orientation. Furthermore, the broadband XRD peaks are substantially wider than those obtained using a monochromatic probe, which prevents to recover quantitative information on the microstructural evolution by FWHM analysis. Finally, a texture function approximating Turneaure's, but refined to ensure qualitative agreement with this earlier work is applied. The misorientation angle is defined as the angle between the ideal direction of the reciprocal lattice vector according to the sample orientation and OR, **G**, and the scattering vector required to reach the pixel, $\mathbf{G}_{pix}$ For misorientations of less than 10° intensity is unaffected, for over 10° the intensity falls as the inverse sixth power of misorientation. It should be noted that we do not attempt to fully recreate the earlier work, but to make only a qualitative comparison for the purpose of confirming how our proposed OR compares to those previously presented by Turneaure. With this model, we obtain a good qualitative agreement between Turneaure's simulation results and ours (Figure S9(a-b)). Some of the features present in Turneaure's experimental data (Figure S9, left panel) are not reproduced by their proposed OR (Figure S9(a-b)). These features can be explained in terms of our proposed OR, either allowing only the variants discussed in the main text, i.e., transition-induced plasticity under uniaxial compression (Figure S9(c)), or by considering all the possible variants, i.e., defect-induced plasticity, in which the growth of the HP phase happens in more hydrostatic conditions and is allowed along every direction (Figure S9(d)).

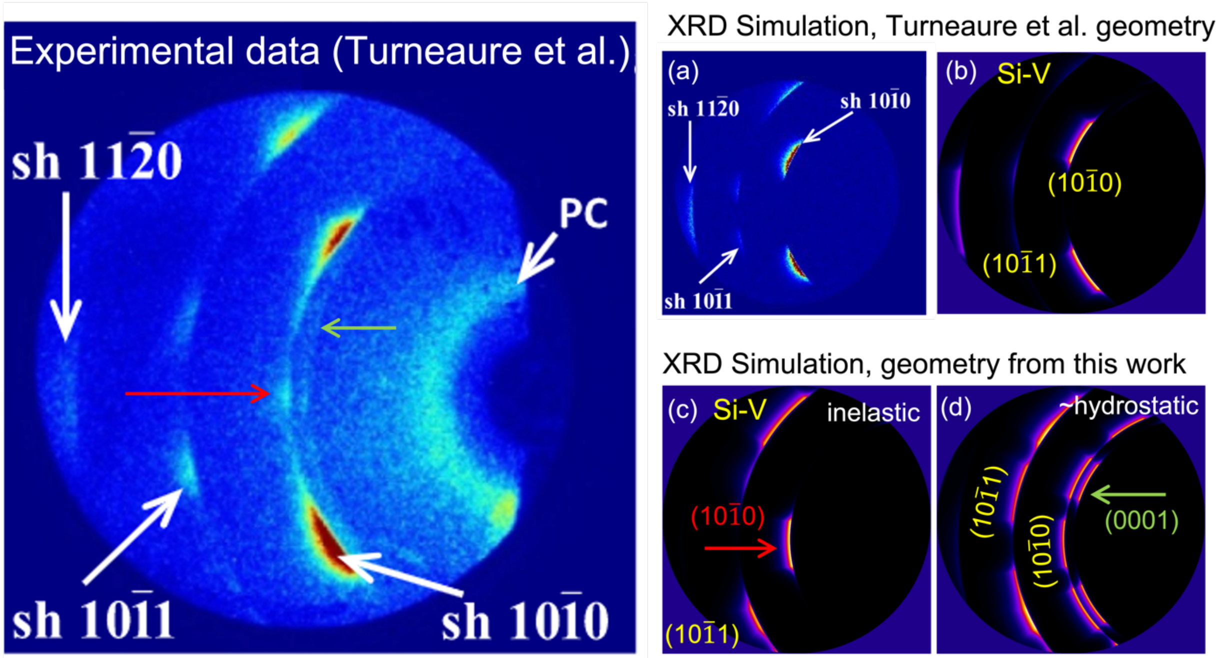

Figure S9. Left: XRD data from Turneaure et al. [11]. Right: XRD simulated patterns with different I-V orientation relationships. (a) simulation from ref. 11; (b) our simulation reproduces the results in ref. 11; (c) simulation using the orientation relationship proposed in the main text, with only the variants allowed under uniaxial compression; (d) simulation using all the possible variants of the orientation relationship proposed in the main text. The red and the green arrow highlight features that are visible in Turneaure's data (left) and not explained in terms of their proposed geometry (a); these features are well reproduced taking into account the orientation relationship proposed here, as shown in (c) and (d).

Left panel and panel (a): Reprinted figure with permission from [S. J. Turneaure, N. Sinclair, and Y. M. Gupta, Physical Review Letters, 117, 045502, 2016]. Copyright 2016 by the American Physical Society."

References:

[1] S. H. Glenzer, L. B. Fletcher, E. Galtier, B. Nagler, R. Alonso-Mori, B. Barbrel, S. B. Brown, D. A. Chapman, Z. Chen, C. B. Curry, F. Fiuza, E. Gamboa, M. Gauthier, D. O. Gericke, A. Gleason, S. Goede, E. Granados, P. Heimann, J. Kim, D. Kraus, M. J. MacDonald, A. J. Mackinnon, R. Mishra, A. Ravasio, C. Roedel, P. Sperling, W. Schumaker, Y. Y. Tsui, J. Vorberger, U. Zastrau, A. Fry, W. E. White, J. B. Hasting, and H. J. Lee, *Matter under Extreme Conditions Experiments at the Linac Coherent Light Source*, J Phys B Atomic Mol Opt Phys **49**, 092001 (2016).
[2] B. Nagler, B. Arnold, G. Bouchard, R. F. Boyce, R. M. Boyce, A. Callen, M. Campell, R. Curiel, E. Galtier, J. Garofoli, E. Granados, J. Hastings, G. Hays, P. Heimann, R. W. Lee, D. Milathianaki, L. Plummer, A. Schropp, A. Wallace, M. Welch, W. White, Z. Xing, J. Yin, J. Young, U. Zastrau, and H. J. Lee, *The Matter in Extreme Conditions Instrument at the Linac Coherent Light Source*, J Synchrotron Radiat **22**, 520 (2015).
[3] H. T. Philipp, M. Hromalik, M. Tate, L. Koerner, and S. M. Gruner, *Pixel Array Detector for X-Ray Free Electron Laser Experiments*, Nucl Instruments Methods Phys Res Sect Accel Spectrometers Detect Assoc Equip **649**, 67 (2011).
[4] L. M. Barker and R. E. Hollenbach, *Laser Interferometer for Measuring High Velocities of Any Reflecting Surface*, J Appl Phys **43**, 4669 (1972).
[5] J. J. MacFarlane, I. E. Golovkin, and P. R. Woodruff, *HELIOS-CR – A 1-D Radiation-Magnetohydrodynamics Code with Inline Atomic Kinetics Modeling*, J Quantitative Spectrosc Radiat Transf **99**, 381 (2006).
[6] O. Strickson and E. Artacho, *Ab Initio Calculation of the Shock Hugoniot of Bulk Silicon*, Phys Rev B **93**, 094107 (2016).
[7] S. P. Lewis and M. L. Cohen, *Theoretical Study of High-Pressure Orthorhombic Silicon*, Phys Rev B **48**, 16144 (1993).
[8] G. Mogni, A. Higginbotham, K. Gaál-Nagy, N. Park, and J. S. Wark, *Molecular Dynamics Simulations of Shock-Compressed Single-Crystal Silicon*, Phys Rev B **89**, 064104 (2014).
[9] M. I. McMahon, R. J. Nelmes, N. G. Wright, and D. R. Allan, *Pressure Dependence of the Imma Phase of Silicon*, Phys Rev B **50**, 739 (1994).
[10] E. E. McBride, A. Krygier, A. Ehnes, E. Galtier, M. Harmand, Z. Konôpková, H. J. Lee, H.-P. Liermann, B. Nagler, A. Pelka, M. Rödel, A. Schropp, R. F. Smith, C. Spindloe, D. Swift, F. Tavella, S. Toleikis, T. Tschentscher, J. S. Wark, and A. Higginbotham, *Phase Transition Lowering in Dynamically Compressed Silicon*, Nat Phys **15**, 89 (2019).
[11] S. J. Turneaure, N. Sinclair, and Y. M. Gupta, *Real-Time Examination of Atomistic Mechanisms during Shock-Induced Structural Transformation in Silicon*, Phys Rev Lett **117**, 045502 (2016).
