# Supplementary material for: Atomistic deformation mechanism of silicon under laser-driven shock compression
Source: Nat Commun. 2022 Sep 21;13:5535. doi: 10.1038/s41467-022-33220-0 (PMC9492784; doi:10.1038/s41467-022-33220-0)
Supplement: Supplementary file 2 — Editorial Assessment Report [file 41467_2022_33220_MOESM2_ESM.pdf]

nature portfolio

GUIDED OPEN ACCESS

nature physics | nature communications | communications physics

Contents of this report

1. **Manuscript details**: overview of your manuscript and the editorial team.
2. **Review synthesis**: summary of the reviewer reports provided by the editors.
3. **Editorial evaluations**: personalized evaluation and recommendation from all 3 journals.
4. **Annotated reviewer comments**: the referee reports with comments from the editors.
5. **Open research evaluation**: advice for adhering to best reproducibility practices.

About the editorial process

Because you selected the **Nature Portfolio Guided Open Access** option, your manuscript was assessed for suitability in three of our titles publishing high-quality work across the spectrum of physics research: ***Nature Physics***, ***Nature Communications***, **and** ***Communications Physics***. More information about Guided Open Access can be found here.

Collaborative editorial assessment

Your editorial team discussed the manuscript to determine its suitability for the Nature Portfolio Guided OA pilot. Our assessment of your manuscript takes into account several factors, including whether the work meets the **technical standard** of the Nature Portfolio and whether the findings are of **immediate significance** to the readership of at least one of the participating journals in the Nature Portfolio Guided Open Access physics cluster.

Peer review

Experts were asked to evaluate the following aspects of your manuscript:

- **Novelty** in comparison to prior publications;
- **Likely audience** of researchers in terms of broad fields of study and size;
- **Potential impact** of the study on the immediate or wider research field;
- **Evidence** for the claims and whether additional experiments or analyses could feasibly strengthen the evidence;
- **Methodological detail** and whether the manuscript is reproducible as written;
- Appropriateness of the **literature review**.

Editorial evaluation of reviews

Your editorial team discussed the potential suitability of your manuscript for each of the participating journals. They then discussed the revisions necessary in order for the work to be published, keeping each journal's specific editorial criteria in mind.

Journals in the Nature portfolio will support authors wishing to transfer their reviews and (where reviewers agree) the reviewers' identities to journals outside of Springer Nature.
If you have any questions about review portability, please contact our editorial office at guidedoa@nature.com.

nature portfolio

GUIDED OPEN ACCESS

nature physics | nature communications | communications physics

Manuscript details

Tracking number	Submission date	Decision date	Peer review type
GUIDEDOA-21-00323	Nov 15, 2021	Jan 13, 2022	Single-blind

Manuscript title

Atomistic deformation mechanism of silicon under laser-driven shock compression

Preprint: https://arxiv.org/abs/2106.06108

Author details

Silvia Pandolfi

Affiliation: SLAC National Accelerator Laboratory, USA

Editorial assessment team

**Primary editor**	**Andrea Taroni**<br>Home journal: ***Nature Physics***<br>ORCID: **0000-0001-9550-5754**<br>Email: **a.taroni@nature.com**
**Other editors consulted**	**Anna Pertsova**<br>Home journal: ***Nature Communications***<br>ORCID: **0000-0002-7831-7214**<br><br>**Daniel Payne**<br>Home journal: ***Communications Physics***<br>ORCID: **0000-0003-3368-2485**
**About your primary editor**	Andrea joined Nature Physics in 2014, having previously worked for Nature Communications and Nature Materials. A graduate of University College London, he completed his PhD in statistical physics. Following a short stint at the École Normale Supérieure de Lyon in France, he undertook postdoctoral work at Uppsala University in Sweden, investigating the dynamics of low-dimensional magnetic systems. Andrea is based in our London office.

Editorial assessment and review synthesis

**Editor's summary and assessment**	The manuscript reports a time-resolved X-ray scattering study of silicon under shock compression. While there is an extensive literature on this system, the contribution of this work is to gain a handle on the atomistic picture of the shear-release mechanism that drives the phase transition under high-pressure.<br><br>From an editorial perspective, a natural home for this study is expected to be *Nature Communications*, since as welcome as the mechanistic insight is, it represents a natural continuation of previous experimental and numerical lines of enquiry, rather than a step-change in understanding that demands broader interest.
**Editorial synthesis of reviewer reports**	The reviewers are generally very positive about the technical quality of this work, and all propose helpful suggestions for strengthening the claims being made.<br><br>In particular, both reviewers #1 and #2 question the distinction being made between "elastic" and "plastic" shear release mechanisms, which we believe goes beyond semantics and requires clarification.<br><br>Although reviewers #1 and #3 explicitly mention *Nature Physics* as a viable outlet for this work, our view is that the arguments put forward by reviewer #2 are compelling: this is clearly very high-quality work, but it perhaps does not reach the very high bar of being seminal. That being said, *Nature Communications* is clearly an achievable outcome for this manuscript.

Editorial evaluations

Nature Physics<br><br>Revision not invited	We do not feel the study matches the criteria for further consideration at *Nature Physics.* This study may well attract interest within the specialist community in high-pressure physics, but given the nature of the advance being presented, the appeal beyond that community appears to be limited.
Nature Communications<br><br>Major revisions	While we acknowledge the influence this study will likely have for the field, we require additional data and analysis in order to consider this manuscript further at *Nature Communications*.
Communications Physics<br><br>Minor revisions	We also appreciate the likely influence of this study among fellow specialists, and are happy to consider a suitably revised study for publication at *Communications Physics*.

Next steps

**Editorial recommendation 1:**	Our top recommendation is to revise and resubmit your manuscript to *Nature Communications.* We feel the additional work required to achieve this would result in a compelling study.
**Editorial recommendation 2:**	You may also choose to revise and resubmit your manuscript to *Communications Physics.* This option might be best if the requested experimental revisions are not feasible at this time.
**Note**	As stated on the previous page *Nature Physics* is not inviting a revision. Please keep in mind that the journal will not be able to consider any appeals of their decision through Guided Open Access.

Revision

To follow our recommendation, please upload the revised manuscript files using **the link provided in the decision letter**. Should you need assistance with our manuscript tracking system, please contact Adam Lipkin, our Nature Portfolio Guided OA support specialist, at guidedOA@nature.com.

Revision checklist

- ☐ Cover letter, stating to which journal you are submitting
- ☐ Revised manuscript
- ☐ Point-by-point response to reviews
- ☐ Updated Reporting Summary and Editorial Policy Checklist
- ☐ Supplementary materials (if applicable)

Submission elsewhere

If you choose not to follow our recommendations, you can still take the reviewer reports with you.

Option 1: Transfer to another Nature Portfolio journal
Springer Nature provides authors with the ability to transfer a manuscript within the Nature Portfolio, without the author having to upload the manuscript data again. To use this service, **please follow the transfer link provided in the decision letter.** If no link was provided, please contact guidedOA@nature.com.

> *Note that any decision to opt in to In Review at the original journal is not sent to the receiving journal on transfer. You can opt in to In Review at receiving journals that support this service by choosing to modify your manuscript on transfer.*

Option 2: Portable Peer Review option for submission to a journal outside of Nature Portfolio
If you choose to submit your revised manuscript to a journal at another publisher, we can share the reviews with another journal outside of the Nature Portfolio if requested. You will need to request that the receiving journal office contacts us at guidedOA@nature.com. We have included editorial guidance below in the reviewer reports and open research evaluation to aid in revising the manuscript for publication elsewhere.

Annotated reviewer reports

The editors have included some additional comments on specific points raised by the reviewers below, to clarify requirements for publication in the recommended journal(s). However, please note that all points should be addressed in a revision, even if an editor has not specifically commented on them.

Reviewer #1 information	
**Expertise**	Materials structures and phase transformations under high pressure
**Editor's comments**	The reviewer is broadly positive, but requests a number of clarifications.

Reviewer #1 comments

Section	Annotated Reviewer Comments
**Remarks to the Author: Overall significance**	This work presents in situ, time-resolved x-ray diffraction study of silicon under laser-driven shock compression. The authors have observed crystallization of high-pressure (HP) phases and formation of a metastable metallic phase upon release, analyzed the microstructural evolution of crystal domains, and provided explanations to observations in experiments under different strain rates. The results provide important insights into the mechanism of silicon deformation under pressure, which supports the inelastic deformation model previously predicted by computer simulations. These original and helpful findings for silicon mark an important step toward understanding the mechanism of materials phase transformations under pressure in general and their dependence on the strength and rate of loading and are of great interest to multiple areas of research and applications.
**Remarks to the Author: Impact**	I believe the paper will be impactful in the field. Nature Communications or Nature Physics might be the most appropriate.
**Remarks to the Author: Strength of the claims**	Overall I think the paper is well written, with a clear and accessible abstract and appropriate introduction and conclusions, and the reported data and methodology are convincing to me as a theorist. I would like to recommend it for publication, provided the authors can improve the clarity by addressing the following questions/comments.<br><br>(major ones:)<br>1. The 12 GPa shot shows crystallization of HP phases as early as 2 ns time delay (Fig.

1 left), but why the 11 GPa shot does not show signatures of any HP phase at 4 ns (Fig. S5)?
2. What are the error bars of the FWHM data in Fig. 4 and the XRD fit in Table S1?
3. plasticity may involve stacking faults or twining, as in metals under bcc-fcc transition, which can consist of crystalline phases. Could this be possible for silicon? If so, would it make the "inelastic" and "plastic" shear release less distinguishable, or even falsify the transition mechanisms proposed here?

The authors should address these major concerns in full.

(minor ones:)
1. an important part of this work is essentially about decompression. It could be better to add in the introduction some discussions about the differences between previous decompression and compression studies.
2. explain the dashed lines for Si-I in Fig. 1.
3. directions of the arrows for the x and y axes for Si-II and z axes for Si-I seem inconsistent with those of the unit cells' edges in Fig. 2a.
4. Fig. 3 upper right panel: shouldn't "Si-V(1-10)" be "Si-V{0-10}", and "()" be "{}"? Are the colored curves and circles all from the calculation or just guides to the eyes?
5. line 97: "V(100)" should be "V(010)"?
6. line 115: "The differences between ..." -- specify what the differences are.
7. Fig. 5b and d: index the phases and planes that correspond to the xrd signals.
8. line 155-158: the description of pressure estimation is not accurate. For example, how do you make the choice when the EOS and hydro simulation give you different pressure values
, such as the negative-pressure case of Si-II in Fig. 1?
9. Supplemental Sec. 2 and 3: references missing for "LLNL's AnalyzeVISAR code" and for "the PROPACEOS software"?
10. Fig. S3: indicate the pressure values for each panel.
11. Fig. S4: the caption is oversimplified; does (a) correspond to ~19 or ~12 GPa, or why not showing the case of ~12 GPa? What is the laser energy for each panel and how do the map change with different laser energies? Also, the range of the color bars may be cut smaller.
12. It would be very helpful to show the 2\theta axis and indicate the locations of the Si-I and 3w signals in the 2\theta-\Phi patterns in Fig. S5--S7.
13. Has any background removal been applied for the Intensity-2\theta plots in Fig. 1, but not in Fig. S5--S7?
14. Table S1: name the last column. Also, at two lines above: "correspond to different pressure values" -- how different?
15. Fig. S7: what does the label "+L" mean? If the sample thickness are not the same for all Runs, better to specify the thickness for each.
16. Supplemental Sec. 6: could you add some discussion about the effect of pink beam? Also, it is not clear what the yellow arrow is pointing to in Fig. S8d.

The authors should also address these minor concerns, and take special care to make reference to the relevant literature in the introduction.

	In addition, I found some typo/wording issues:<br>line 8: "a ... diagrams"<br>line 38: "whole-profile fit" might need some elaboration or a reference?<br>line 40: "The intensity of the XRD signal from the HP phases increases with time" -- this is not an exact description for all signals of the HP phases.<br>line 137: "picosend".<br>Captions of Fig. S5--S6: "form the 3w beam".<br>Supplemental Sec. 6: "falls of as".<br>Captions of Fig. S8: "or=f".
**Remarks to the Author: Reproducibility**	N/A

Reviewer #2 information

**Expertise**	Numerical simulations of matter under high pressure
**Editor's comments**	The reviewer appreciates the quality of the study, although they question the strength and the novelty of the mechanism being reported.

Reviewer #2 comments

Section	Annotated Reviewer Comments
**Remarks to the Author: Overall significance**	The paper reports a shock wave study on crystalline silicon.<br><br>I was immediately confused by the title - I don't know what the difference is between plastic and inelastic deformation - I would call both inelastic. Perhaps phase transition induced plasticity would be clearer. It is misleading to call it deformation when after the deformation it is no longer in the parent phase. Perhaps the claim is based on the fact that the materials returns to Si-I on decompression?<br><br>**This comment echoes, albeit more forcefully, a concern raised by reviewer #1. The authors are strongly encouraged to better articulate the distinction between plastic and inelastic deformation, and consider this reviewer's suggestion to use a different terminology.**

	A single crystal is studied with X-ray diffraction pulsed at a nanosecond timescale to observe the change in pattern as the wave passes through.<br>The authors report that the silicon undergoes a transformation from diamond Si-1 to known high-pressure phases Si-V and possibly Si-XI: beta-Sn Si-11 is observed on decompression.<br><br>I don't see any evidence for Si XI in the XRD patterns in the paper or SM. Only 3-4 peaks are visible. The SM XRD figures are perhaps the most useful for review, but they are incomplete. For readers who don't carry the XRD pattern of all silicon phases in their heads, it would be helpful if the authors could, for reference, include the ideal powder pattern for Si II, V and XI. At the moment there is no indexing or tagging of peaks except Si-I<br><br>**The authors should attempt to address this concern by providing the additional data and information being requested.**<br><br>The evidence for the orientation of the Si-V relative to the initial Si-I is convincing. It looks to me like the Si-V is twinned. The orientation relation is strong evidence that the transformation occurs directly from I-V as claimed.<br><br>Si-XI appears only in fig 1c. I find it very inconclusive, there will surely be huge shear strains in the crystallites which could account for the signal.
**Remarks to the Author: Impact**	The paper provides evidence of phase transition induced plasticity in shock. It is a fairly well established phenomenon. The claimed novelty is that the material is non-metallic, however the high pressure phases are all metallic, so this is only partially true.<br><br>The result is nice, but I don't find it especially surprising. Plasticity is difficult in the covalently bonded Si-I, so it transforms to the HP metallic phase. In this big picture II, V, and XI are similar open metallic phases, and the fact that they deform plastically more easily than phase I is no surprise. I think all this would be expected based on static work, although confirmation is nice.<br><br>There are many examples of transformation under shock from single crystal to specifically oriented HP phases which determine the transition path. This work is nice, but not seminal.<br><br>The recovery of phase I on decompression is interesting: it doesn't happen under static compression. It would be interesting to understand why: is it because we are at high enough temperature that the ground state can be easily accessed, or is the decompression so fast that the stability regimes of the ST12/BC8 phases is bypassed. Presence of some Si-I is proved by XRD, but how is the claimed absence of amorphous silicon determined?<br><br>**While not essential for further consideration of the manuscript, it**

	**would be helpful to address this question if possible.**
**Remarks to the Author: Strength of the claims**	As stated above, I'd like to see a better description of the patterns shown in the SM, and more convincing evidence for Si-XI. However, my strong suspicions is that the data shows just what they claim.<br><br>Overall, I'm convinced this is a nice study which should be published with minor revisions. There are so many Nature journals these days I can't keep up with which is most appropriate.<br><br>**In view of the other reviewers' comments and journals' own criteria, the editors interpret this comment as an endorsement for either *Nature Communications or Communications Physics.***
**Remarks to the Author: Reproducibility**	This study is easily reproducible if one has access to LCLS, or other similar facilities.

Reviewer #3 information

**Expertise**	Dynamically-compressed matter, time-resolved spectroscopy
**Editor's comments**	The reviewer is broadly positive, but makes a number of requests for clarification.

Reviewer #3 comments

Section	Annotated Reviewer Comments
**Remarks to the Author: Overall significance**	The authors of the present manuscript studied shock-compressed silicon making use of the time-resolution of the LCLS XFEL. Si has been the subject of several molecular dynamics (MD) simulations that have proposed two competitive mechanisms to model the transformation at high-pressure under high strain rate. Using the x-ray diffraction (XRD) probe on a 100-oriented single crystal of Si under laser-shock compression they claim to resolve this transformation mechanism.
**Remarks to the Author: Impact**	The subject is important, as it is a significant test for the MD simulations – even more seeing that the simulations are getting closer to the timescales of experiments. The use of a 60 fs x-ray beam provided by the XFEL appears very

	well suited for the study of this type of phenomenon. The manuscript is well written and reference previous literature appropriately. While this is not the first time that XRD is used to study dynamically compressed Si, this is the first time under laser-shock compression on a single crystal at the FEL. The results presented in this manuscript may have a significant impact on a broad readership and could potentially offer some interesting ideas to explain the phase lowering transitions that are currently being observed in a lot of material (Si, Sb, Bi…) under dynamic compression.
**Remarks to the Author: Strength of the claims**	The work is convincing , however there are some details and questions that need to be addressed/clarified.<br><br>1 - Some clarifications of the transformation mechanism seems to be needed. Line 79, the authors state they use the mechanism proposed by MD simulations from ref 38. However, the high-pressure structure determined from MD simulations for shocked Si(100) was identified as Imma while sh-Si is observed in this case. Moreover, the MD simulations from this reference have predicted a mixed state at the peak pressure: one cannot really know if this mixed state as an homogenous pressure state is never probed when looking at figure S4. Additionally, reading line 81 Si-II appears as an intermediate phase before shear-stress releasing towards Si-V. Could the authors clarify why Si-II is not seen upon compression? Could have smaller time steps (or peak pressure) been used or perhaps the large jitter would make it irrelevant?<br><br>**This comment seems especially pertinent, and the authors are encouraged to elaborate on the connections that can be drawn between theory and experiment here.**<br><br>2 - This leads to another comment: one would like to know about the reproducibility from shot to shot.<br><br>3- Ref 39 starts to observe liquid-Si below ~0.7 V/V0 which doesn't seem to be the case in your data. How do you explain this difference?<br><br>4 - Why did you limit the starting material to Si(100) and did not try other orientations?<br><br>5 - The Φ (phi) coverage is rather small. Looking at the ref 39 there seems to be additional CSPAD available in this study. Could they not be used to have a better coverage?<br><br>6 - Figure 1: I would have liked to see the reference material in (a) and (d) before the 2ns shot.<br>Looking at the dashed lines for Si-I it seems like it is decompressing from the start?<br>(d) 20.5ns you state in the supplementary that the starting material has

	changed: maybe put it directly in the main text and explain how it changed.<br>(a) 21.3ns: there are two additional peaks around 35 and 50°. Even though they do not seem relevant, could you comment?<br><br>7 - Figure 3: According to your model the peak that is observed are Si-V(1-10) yet you have labelled your peaks as Si-V(010) in figure 1. Is this a mistake or is there something I did not fully understand? I guess that if it is the case it should probably made clearer.<br><br>8 - Figure 5: Labelling the peaks would help. Also, since you are comparing with ref 41, using the 4 Miller-Bravais index would probably be better. It would be more interesting to be able to directly compare XRD signature with your XRD patterns.<br><br>9 - Comparison with ref 41: Could the mechanism also be applied for other orientations, like the (111) that was done in ref 41? I do not fully agree with you that the mechanism from the gas gun experiment only results from defect plasticity. From your simulations and fig S8, it seems rather that is a mix between ref 41 and the inelastic mechanism.<br><br>10 - Supplementary information:<br>I am no expert in VISAR analysis, but I was wondering if it is normal that the free surface starts to move past 10ns in the 43microns sample presented in fig S3 but the shock break out is around 5.5ns in the simulation. Was the fluence used different?<br>Fig S4: the pressure scale is too high, it would probably be better to limit it at <25 GPa to have a better resolution.<br>Show Lebail profile refinements. Note that doing a refinement on two peaks is not very relevant. Hence having 3 significant digits in table S1 does not seem reasonable.<br>Add the theta and phi scales on fig S5-6 and 7.
**Remarks to the Author: Reproducibility**	good

Open research evaluation

General information

Guidelines for Transparency and Openness Promotion (TOP) in Journal Policies and Practices ("TOP Guidelines")

The recommendations and requests in the table below are aimed at bringing your manuscript in line with common community standards as exemplified by the TOP Guidelines. While every publisher and journal will implement these guidelines differently, the recommendations below are all consistent with the policies at Nature Portfolio. In most cases, these will align with TOP Guidelines Level 2.

FAIR Principles

The goal of the recommendations in the table below related to **data or code** availability is to promote the FAIR Guiding Principles for scientific data management and stewardship (*Scientific Data* **3**: 160018, 2016). The FAIR Principles are a set of guidelines for improving 4 important aspects of digital research objects: **F**indability, **A**ccessibility, **I**nteroperability and **R**eusability.

ORCID

ORCID is a non-profit organization that provides researchers with a unique digital identifier. These identifiers can be used by editors, funding agencies, publishers, and institutions to reliably identify individuals in the same way that ISBNs and DOIs identify books and articles. Thus the risk of confusing your identity with another researcher with the same name is eliminated. **The ORCID website** provides researchers with a page where your comprehensive research activity can be stored.

Springer Nature collaborates with the ORCID organization to ensure that your research contributions (as authors and peer reviewers) are correctly attributed to you. Learn more at https://www.springernature.com/gp/researchers/orcid

Specific advice

Data availability
**Data Availability Statement**
Thank you for including a Data Availability Statement. In order to adhere to community standards for transparency and reproducibility, we encourage you to amend this so as to make it clear which data are available and under what conditions.<br><br>Please find more information about Data Availability Statements and Springer Nature's data policies here.

Code availability and citation
To adhere to community standards and promote transparency in research, we encourage the Code Availability Statement to indicate whether and how the code or algorithm can be accessed, including any restrictions to access. Public release of custom software may be required for publication in a Nature Portfolio journal.<br><br>Upon publication, Nature Portfolio journals consider it best practice to release custom computer code in a way that allows readers to repeat the published results. Code should be deposited in a DOI-minting repository such as Zenodo, Gigantum or Code Ocean and cited in the reference list following the guidelines described in our policy pages. Authors are encouraged to manage subsequent code versions and to use a license approved by the open source initiative. Full details about how the code can be accessed and any restrictions must be described in the Code Availability statement.<br><br>We also provide a Code and Software submission checklist that you may find useful.
**Reporting & reproducibility**
All source data underlying the graphs and charts presented in the main figures must be made available as Supplementary Data (in Excel or text format) or via a generalist repository (eg, Figshare or Dryad). This is mandatory for publication in a Nature Portfolio journal, but is also best practice for publication in any venue.<br><br>The following figures require associated source data: Fig. 4a-f.
Nature Portfolio journals allow unlimited space for Methods. The Methods must contain sufficient detail such that the work could be repeated. It is preferable that all key methods be included in the main manuscript, rather than in the Supplementary Information. Please avoid use of “as described previously” or similar, and instead detail the specific methods used with appropriate attribution.
